# Prolactin-secreting tumors, dopamine agonists and pregnancy: a longitudinal experience of a tertiary neuroendocrine center

**DOI:** 10.1007/s11102-024-01384-1

**Published:** 2024-03-18

**Authors:** Nunzia Prencipe, Chiara Bona, Daniela Cuboni, Alessandro Maria Berton, Fabio Bioletto, Emanuele Varaldo, Luigi Simone Aversa, Michela Sibilla, Valentina Gasco, Ezio Ghigo, Silvia Grottoli

**Affiliations:** 1 Department of Medical Science, Division of Endocrinology, Diabetes and Metabolism, University of Turin, Corso Dogliotti 14, 10126 Turin, Italy; 2 Division of Endocrinology, Diabetology and Metabolism, S. Croce and Carle Cuneo Hospital Districts, Cuneo, Italy

**Keywords:** Lactation, Delivery, Cabergoline, Bromocriptine, Prolactinoma

## Abstract

**Purpose:**

Prolactin (PRL)-secreting tumours are associated with infertility and can be reverted by dopamine agonist (DA) therapy. The suspension of DA is recommended once pregnancy is established, as all DAs cross the placenta. The aim of the study was to evaluate the rate of maternal-foetal complications in women treated with cabergoline (CAB) or bromocriptine (BRM) for prolactinoma during gestation and the effect of pregnancy on prolactinoma progression.

**Methods:**

This was a retrospective observational study involving 43 women affected by prolactinoma who became pregnant during therapy with CAB or BRM for a total of 58 pregnancies. For each patient, medical records were analysed by integrating the data with outpatient or telephone interview.

**Results:**

At the time of conception, 18 women were in the BRM group, while 40 were in CAB group. No differences were found in obstetric or neonatal outcomes between the two groups. There was a significant difference (p = 0.046) in child complications reported in maternal interview found exclusively in the CAB group. No further confounding factors were detected. Disease remission rate after the first pregnancy was 42.9% and the main predictor was a lower PRL nadir before pregnancy (p = 0.023). No difference was detected between the two groups in terms of tumor remission. Breastfeeding did not modify the outcome.

**Conclusion:**

Foetal exposure to DAs during the first weeks of embryogenesis is not associated with a greater risk of complications. The transient and mild developmental disorders recorded resolved spontaneously and the prevalence was substantially overlapping with that observed in the general population.

**Supplementary Information:**

The online version contains supplementary material available at 10.1007/s11102-024-01384-1.

## Introduction

Prolactinomas represent the most common cause of hyperprolactinemia and account for 40% of all pituitary adenomas [1]. Prolactinoma has a prevalence of 60–100 cases/million, and it is more common in women, especially those aged 20 to 50 years [2]. In women, hyperprolactinemia is a frequent cause of anovulatory cycles and infertility, but fertility can often be restored by treatment with dopamine agonists (DAs) [3]. DA represents the first-line therapy since it regulates menses and can reduce pituitary lesion size, restoring normal PRL levels [4]. During DA therapy, 85% of women desire offspring. However, there are some significant concerns regarding the treatment of prolactinomas with DA during conception and pregnancy. The literature has extensively demonstrated that, compared with the general population, bromocriptine (BRM) does not increase the number of miscarriages, premature births, multiple pregnancies or congenital malformations [3, 5, 6]; moreover, available information on the safety of cabergoline (CAB) is reassuring, although specific data remain scant [3, 7–9]. Despite the demonstration that DAs used previously and during pregnancy are safe drugs, the Endocrine Society recommends the immediate suspension of medical therapy once pregnancy is confirmed as a precaution [4].

The latest Pituitary Society guidelines also recommend withdrawal of DAs for patients with microprolactinomas and intrasellar macroprolactinomas as soon as pregnancy is confirmed. However, they allow for the possibility of continuing DA therapy in the case of expansive/invasive macroadenoma [10].

As regards the Italian regulation, cabergoline should even be interrupted once ovulatory cycles have been re-established, at least one month before the supposed conception, or immediately, at the time of ascertained pregnancy. The possibility of stopping treatment once pregnancy is established derives from a limited series of 329 pregnancies, suggesting that foetal exposure to cabergoline through early pregnancy does not induce any increase in the risk of miscarriage or foetal malformation [11].

Another relevant aspect is the possible impact of pregnancy and breastfeeding on the progression of prolactinoma. In fact, some studies have shown that 10–68% of women with prolactinoma or idiopathic hyperprolactinemia experience disease remission following pregnancy. Breastfeeding also does not seem to increase the risk of persistence or worsening of the disease [12–15].

Based on these data, the purposes of the present study were 1) to evaluate the prevalence of short- and long-term maternal or foetal complications in patients with prolactinoma treated with CAB or BRM during pregnancy induction or during pregnancy itself, afferent to the Neuroendocrinology Centre of A.O.U. City of Health and Science of Turin; and 2) to evaluate the effect of pregnancy on the remission of hyperprolactinemia and on the progression of prolactinoma.

## Methods

This was a retrospective single-center longitudinal observational study involving a cohort of women affected by prolactinoma who were followed up at the Neuroendocrinology Centre of University Hospital City of Health and Science of Turin (Italy) and who became pregnant during treatment with CAB or BRM. Women with hyperprolactinemia caused by causes other than prolactinoma were not included in the present study. All pregnancies that occurred from 1981 to 2012 were taken into consideration to allow a postpartum follow-up of at least ten years to assess any late side effects. Clinical and hormonal data are shown in Table 1.

**Table 1:** Differences between clinical and biochemical data from patients with cabergoline- and bromocriptine-induced pregnancies

Variables	Pregnancies on Cabergoline (n. 40)	Pregnancies on Bromocriptine (n. 18)	P
Age at diagnosis (years); mean ± SD	27.4 ± 6.1	26 ± 5.7	0.413
Age at pregnancy (years); mean ± SD	33.2 ± 5.4	30.7 ± 5.1	0.111
Microadenoma; n (%)	34 (85%)	12 (67%)	0.161
Adenoma maximum diameter at diagnosis (mm); median [IQR]	6 [5.3; 8]	6.5 [5; 12]	0.919
Prolactin levels at diagnosis (ng/ml); median [IQR]	110.1 [62.4; 143]	112.3 [36; 318]	0.579
Adenoma diameter nadir before pregnancy (mm); mean ± SD	4.9 ± 3	7.3 ± 3.7	**0.023**
Prolactin levels nadir before pregnancy (ng/ml); median [IQR]	11 [4.5; 22.1]	5 [3.7; 19.5]	0.550
Breastfeeding (yes); n (%)	25 (64%)	10 (59%)	0.776
Breastfeeding weeks; median [IQR]	11 [0;35]	4 [0;16]	**0.040**
DA foetal exposure (days); median [IQR]	28 [28;35]	35 [28;35]	0.378
Preterm deliveries; n	2	6	0.812
Child weight at birth (gr); median [IQR]	3400 [3240; 3750]	3400 [3040; 3647]	0.600
Child born underweight; n (%)	3 (7.5%)	2 (11.2%)	0.892
Child born with macrosomia; n (%)	4 (10%)	1 (5.5%)	0.916
APGAR Score; median [IQR]	9 [9; 9]	9 [9; 9]	0.848
Child gender male; n (%)	22 (61%)	9 (56%)	0.767
Persistence of disease; n (%)	26 (67%)	11 (61%)	0.768
Adenoma diameter at last follow up (mm); median [IQR]	2 [0;5.2]	3 [2;5.5]	0.247
Prolactin levels at last follow up (ng/ml); median [IQR]	14.1 [8.7; 39.9]	20.1 [5.7; 48.6]	0.686
Delivery/pregnancy complications (yes); n (%)	11 (28%)	3 (18%)	0.513
Child complications (yes); n (%)	9 (24%)	0 (0%)	**0.046**
Child speech disorders (yes); n (%)	6 (15%)	0 (0%)	0.167
Duration of follow-up (years); mean ± SD	12.7 ± 6.3	24.7 ± 6.3	** < 0.01**

*SD* standard deviation; *IQR*  interquartile range; *DA* dopamine agonist

The diagnosis of prolactinoma was made according to the criteria of the International Guidelines [4, 10], while disease remission was defined as persistence of biochemical and clinical normalization at least 1 year after therapy withdrawal.

For each patient, computerized medical records were analysed; data were also integrated through an outpatient visit or, in the absence of this possibility, by telephone interview. The salient clinical and pregnancy-related data (outcome, type of delivery, complications, breastfeeding), drug exposure (duration of exposure to CAB/BRM, starting dose, maximum dose) and development of the child (birth complications, weight at birth, APGAR score, malformations, developmental deficiencies) were evaluated. The data regarding the course of pregnancy, delivery, and the child's health conditions were collected through interviews with the study patients and not through direct access to the gynecological or pediatric clinical records (patient interview script available on Supplemental Material).

Data on PRL levels at diagnosis, the nadir of PRL levels before pregnancy and at the last follow-up were included as adenoma features at the same time. The study was conducted according to the principles of the Declaration of Helsinki. All patients signed an informed consent form for study participation. The study was approved by the Internal Ethical Committee of our institution.

### Statistics

The baseline characteristics of all patients included in the analysis are summarized using medians and interquartile ranges (IQRs) for continuous data (or means and standard deviations when specified) and rates and percentages for binary and categorical data. Between-group differences in personal and clinical features at diagnosis were evaluated by Student’s t test, the Mann‒Whitney U test, ANOVA and the Kruskal‒Wallis test for continuous variables. For categorical variables, the chi-square test or Fisher’s exact test was applied, where appropriate, considering normality with the Shapiro‒Wilk test and the number of independent groups. Univariate logistic regression analysis was subsequently performed using the "MedCalc™" program version 18.11.3. The results were considered significant at p < 0.05.

## Results

Forty-three patients were studied (age at diagnosis 27 ± 6.0 years and age at pregnancy 32.4 ± 5.4 years) for a total of 58 pregnancies (31 patients underwent one pregnancy; two pregnancies were recorded in nine patients, and three were recorded in three patients). Among the 43 women studied, 10 (23.3%) had macroadenomas, and 33 (76.7%) had microadenomas. At diagnosis, the median tumor diameter was 6 mm (IQR 5–9 mm), and the median PRL was 112.3 ng/ml (IQR 50–149.7 ng/ml). None of the patients underwent pituitary neurosurgery (NS) or radiotherapy (RT) before pregnancy, while a single patient underwent NS during the postpregnancy follow-up (six years later). By the time of conception, 18 patients were receiving BRM therapy (dose 6.16 ± 6.6 mg/day; IQR 2.5–20 mg/day), and 40 were receiving CAB (dose 1.6 ± 3.3 mg/week, IQR 0.25–17.5 mg/week). The two populations analysed were comparable in terms of age at diagnosis, age at pregnancy, prevalence of macro/microadenoma, adenoma size at diagnosis and PRL levels at diagnosis (Table 1). The estimated cumulative dose of BRM taken during pregnancy was 274.8 ± 399 mg (IQR 2.5–1260 mg), the median foetal exposure time was 35 days (IQR 28–35 days), and the estimated cumulative dose of CAB was 4.6 ± 4.3 mg (IQR 1–18 mg), with a median foetal exposure time of 28 days (IQR 28–35 mg) (Table 1**)**. Although all patients were instructed to interrupt DA therapy once pregnancy was confirmed, in two cases, the patients continued BRM therapy throughout the course of gestation (in one there was no medical indication; in the other, there was severe headache). The patients were followed up for at least 10 years, with a mean of 16.4 ± 8.4 years (CAB 12.7 ± 6.3 vs BRM 24.7 ± 6.3; p < 0.01).

### 1) Effectiveness of DA therapy before conception

**Fig. 1:**
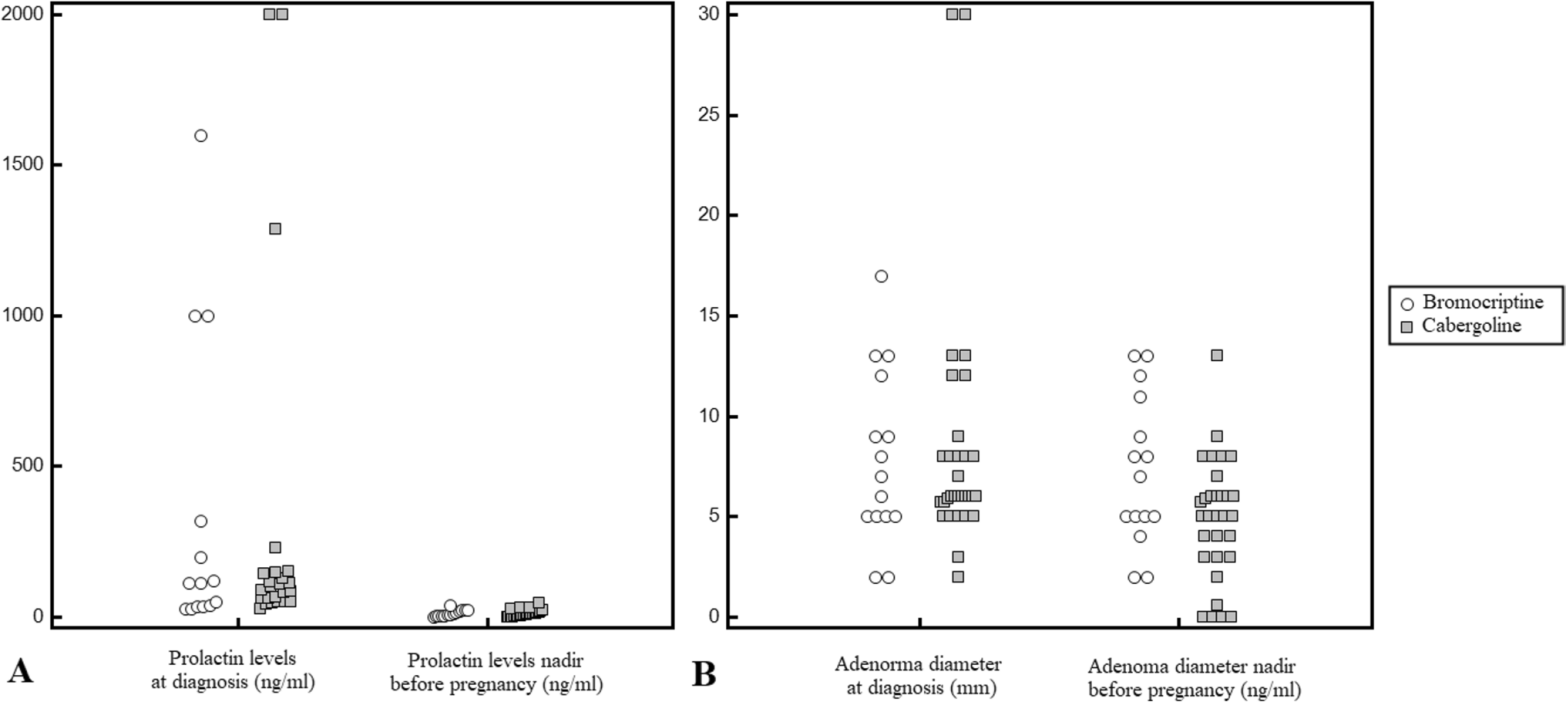
**A**: Prolactin levels (ng/ml) at diagnosis and before pregnancy. **B**: adenoma maximum diameter (mm) at diagnosis and before pregnancy

Thirty-three of 43 patients (76.7%) achieved normalization of PRL levels before pregnancy (nadir vs pretreatment: 9.5 ng/ml, IQR 4.3–20.5 ng/ml vs 112.3 ng/ml, IQR 50–149.7 ng/ml; p = 0.0003; Fig. 1A); of these, 15 patients had inhibited PRL levels (< 4.8 ng/ml). There was no significant difference between CAB and BRM in terms of treatment duration to achieve PRL normalization, although patients treated with CAB tended to normalize PRL levels earlier (4.3 ± 1.7 months vs 9.4 ± 12.3 months; p = 0.08). In 11/43 patients (25.6%), the pituitary lesion exhibited a significant (> 20%) diameter reduction (4.9 ± 3.0 mm vs 8 ± 5.45 mm; p = 0.01; Fig. 1B), and in three patients, the lesion completely disappeared before pregnancy. In all the other patients, the lesion did not undergo volumetric variations. The diameter of the tumor before pregnancy was significantly lower in the CAB group (p = 0.023) (Table 1).

### 2) Obstetric and neonatal outcomes

Forty-eight pregnancies (82.7%) came to term (38th–42nd week), eight ended in a preterm delivery (two in the CAB group and six in the BRM group), and two ended in a spontaneous abortion (both at the 10th week, one in the CAB group and one in the BRM group) (Table 1). No congenital malformations, gestational trophoblastic disease or extrauterine implantation was found in any of the pregnancies. Sixteen of the 56 (28.6%) deliveries occurred by caesarean section (six cases for breech presentation, one case for macrosomia in gestational diabetes, one case due to absence of valid contractions, one case for wrapping of the umbilical cord around the neck, three cases due to the advanced age of the mother, two cases for foetal distress and two for preeclampsia; *supplemental material: *Table 1S), 38/56 pregnancies (67.8%) resulted in spontaneous deliveries, and two were pharmacologically induced. The median new-born weight at birth was 3400 g (IQR 3240–3750 g), and the median APGAR score was 9 (IQR 9–9). In the population of women receiving BRM, two children were born underweight (weight < 2500 g) (11.2%), 14 had a normal weight (77.8%) and one had macrosomia (5.5%); on the other hand, in the population of women treated with CAB, three children were found to have a weight at birth < 2500 g (7.5%), 32 had a normal weight (80%), and four had a macrosomal weight (10%). However, no significant difference was found in child weight between the CAB and BRM treatment groups (Table 1).

The following complications occurred during pregnancy or delivery: two cases of preeclampsia, two cases of gestational diabetes, one case of placenta previa and a new diagnosis of uterine fibroma. Foetal distress problems occurred in seven patients who presented with acute respiratory distress syndrome (ARDS) (Table 1, Table 1S).

Two of the patients studied continued to take BRM for the entire duration of gestation: the first gave birth preterm by caesarean section but had no complications in terms of foetal outcome; the other patient delivered spontaneously at full term without any complications during child development.

Thirty-six of the 56 infants (64.3%) were breastfed for a mean duration of 15.56 ± 20.32 weeks (range 2–96 weeks). The duration of breastfeeding in CAB patients was significantly longer (p = 0.04) (Table 1).

The two groups showed a slight difference in terms of child complications during follow-up; these complications were more frequent (p = 0.046) in the CAB group (22.5% of CAB pregnancies, 15.5% of all pregnancies). To reduce bias resulting from including siblings, we performed a subanalysis solely on the first pregnancies and no statistical difference was confirmed, although a trend of significance persists (*supplemental material: *Table 2S).

In women treated with CAB at conception, speech disorders (transient) were found in six children, walking delay in one case and atopic dermatitis during childhood in two cases. None of these were found in the BRM women (Table 1)*.*

According to the descriptive statistics, no significant differences were found in terms of obstetric (complications during pregnancy, characteristics of delivery, spontaneous/caesarean delivery, or full/preterm birth) or neonatal (APGAR, weight at birth, malformations) outcomes between the CAB and BRM groups (Table 1).

Given that developmental disorders (speech and walking delay) were the most common alterations observed in children, we investigated any predictors of these disorders by assessing both maternal and foetal factors. Univariate regression analyses revealed that maternal age at pregnancy, weight at birth, APGAR score, breastfeeding duration, days of DA exposure, and pregnancy/delivery complications did not interfere with developmental problems (Table 2). Analysing these data considering first pregnancy only (data available for 41 patients) no significant differences emerged *(supplemental material: Table 3S).*

**Table 2:** Variables associated with developmental disorders

Variables	No developmental disorders (n. 46)	developmental disorders (n. 7)	P
Age at diagnosis	26.6 ± 6.0	29.0 ± 7.1	0.351
Age at pregnancy	32.1 ± 5.3	34.1 ± 7.1	0.379
Microadenoma; n (%)	36 (78%)	6 (86%)	0.962
Adenoma maximum diameter at diagnosis (mm); median [IQR]	7 [5;9]	6 [6;6]	0.731
Prolactin levels at diagnosis (ng/ml); median [IQR]	121 [53–221]	65 [48.5–87.7]	0.097
Adenoma diameter nadir before pregnancy (mm); median [IQR]	5.8 [4.5–8.0]	4.0 [0.0–6.0]	0.096
Prolactin levels nadir before pregnancy (ng/ml); median [IQR]	10.2 [4.7;21.2]	4.5 [0.9;19.4]	0.265
Breastfeeding (yes); n (%)	29 (63%)	5 (71%)	0.994
Breastfeeding duration (weeks); median [IQR]	4 [0–24]	2 [0–39]	0.669
Foetal Exposure to DA (days); median [IQR]	28 [28–35]	28 [28–35]	0.770
CAB; n (%) BRM; n (%)	31 (67.4%) 15 (32.6%)	7 (100%) 0 (0%)	0.171
Child weight at birth (gr); median [IQR]	3350 [3062–3692]	3530 [3295- 3652]	0.874
Delivery/pregnancy complications (yes); n (%)	11 (24%)	2 (29%)	0.937
APGAR SCORE; median [IQR]	9 [9–9]	9 [9–9]	0.205
APGAR SCORE			0.386
≤ 6	0	0	
7	1	0	
8	1	1	
9	26	6	
10	6	0	

*IQR* interquartile range; *SD* standard deviation, *DA* dopamine agonist

### 3) Effect of pregnancy and breastfeeding on hyperprolactinemia and pituitary adenoma

After the last pregnancy, in 21/43 women (48.8%), remission of hyperprolactinemia, defined as normalization of PRL levels, was documented in the absence of therapy, while disease persistence was found in 22/43 (51.2%). Unexpectedly, in our series, a greater number of pregnancies per patient seemed to be associated with the persistence of the disease (p = 0.04).

To assess the impact of pregnancy on the pituitary lesions, we exclusively considered the first pregnancy of each patient (data available for 42 pregnancies). This approach was employed to eliminate the potential influence of restarting DA therapy between successive pregnancies. After the first pregnancy, 42.9% of patients achieved biochemical remission, while 57.1% exhibited persistent disease. Specifically, among those who achieved remission, 11/28 patients (39%) were treated with CAB, while 7/14 (50%) with BRM. However, no statistically significant difference was observed (p = 0.740).

The two populations were similar in terms of age at diagnosis (p = 0.336), age at pregnancy (p = 0.354), prevalence of macro/microadenoma (p = 0.875), size of the adenoma at diagnosis (p = 0.574), breastfeeding (p = 0.920), and duration of breastfeeding (p = 0.633). PRL levels at diagnosis were similar between the two populations (p = 0.405), while the prepregnancy PRL nadir was significantly lower in patients who achieved biochemical remission (4.6 ng/ml, IQR 2.8–10.6 ng/ml vs 12.4 ng/ml, IQR 4.8–27.0 ng/ml; p = 0.023).

Among the patients who did not achieve remission, 17 restarted CAB, four restarted BRM, and three did not receive DA therapy. At the last follow-up, adenoma data were available for 22 of these patients. In the CAB group, four patients maintained lesion stability, nine experienced a reduction, and two experienced complete shrinkage. In the BRM group, two patients had stable lesions, one had a reduction, one had an increase, and none had total shrinkage. Nevertheless, these differences were not statistically significant (p = 0.134).

## Discussion

Our study confirmed the safety of administering DA to women affected by PRL-secreting tumours during the first weeks of pregnancy for maternal and foetal outcomes and for pituitary adenoma. In fact, in our series, the incidence of abortion was lower than that in the general population (6–9%) [16] or compared to that in other studies conducted on a similar population (9.1%, 9.8%, 10.2%, respectively) [11, 17, 18]. Furthermore, no cases of malformations have been reported in children born to women treated with BRM or CAB, in agreement with the findings of previous studies [18]. Additionally, the percentage of preterm births was comparable to that of the general population (13.7% vs 12.7%), as was the percentage of low-birth-weight children (8.6% vs 8% in the general population) [19]. Additionally, in the case of a woman who was taking BRM during the whole pregnancy, no complications were detected.

No statistically significant differences were found in terms of obstetric or neonatal outcomes between CAB and BRM treatment. However, a higher rate of child complications (six speech disorders, one walking delay, two atopic dermatitis) in infants born was found in the CAB group. Specifically, among them, developmental disorders (walking and speech delay) constituted the majority. All were mild and transient, and they were not influenced by the mother's age, birth weight, or breastfeeding. Although language disorders did not significantly differ between the two groups, in our case study they occurred in 10.7% of the whole children population (all in the CAB group). Also Lebbe et al. investigated this aspect in a series of 100 pregnancies and found no difference in verbal fluency [15]. Furthermore, the reported prevalence of specific language impairments in the general population varies widely from 2 to 12% [20]. In our study, a 15% (6/40) prevalence was documented in the CAB group,10.7% when considering the whole population studied, which is consistent with the findings of the literature. Very little is known about the etiology, and multiple causes are probable. In her work, Diepeveen et al. concluded that children with a speech disorder tended to have lower APGAR scores 5 min after birth; in addition, the difference in APGAR scores was greater for females than for males [20]. In our study, no difference emerged in the APGAR score between the two groups.

Our data demonstrate the safety of DAs in pregnancy, in contrast with the data from the EFEMERIS pharmaco-epidemiological database study, which showed an increase in the rates of abortion and premature birth [21]. The data from this specific study were evaluated by the authors of the recent Pituitary Society guidelines [10]. In addition, most women in the exposed groups were treated with BRM and other DAs (quinagolide, lisuride, ropinirole, and piribedil) but not CAB. Conversely, a review of the literature for the European Society of Endocrinology Clinical Practice Guidelines, in accordance with our data, showed normal rates of miscarriage, preterm delivery, and congenital malformations with the use of CAB in prolactinoma [22].

The second endpoint of our study was to evaluate the impact of pregnancy and breastfeeding on pituitary tumours. Our results are less optimistic than previous ones. Indeed, the postpregnancy remission rate was 42.9%, which was significantly lower than the 68% reported by Auriemma et al. [14]. A longer medical treatment before pregnancy and the inclusion of women with functional and nontumour hyperprolactinemia are possible causes of this discrepancy. In fact, in our study, we included only women with pituitary lesions and excluded patients with idiopathic hyperprolactinemia. On the other hand, the remission rate reported in our study was higher than that reported in other previous works, in which remission was documented in 15 to 35% of hyperprolactinemic women [23–25]. Notably, in our study, we found no significant differences between women taking BRM or CAB. A higher probability of disease remission is related to lower PRL nadir obtained before pregnancy, which probably implies that the response to DA therapy plays a greater role in disease remission than pregnancy. We did not observe any correlation between the size of the adenoma at diagnosis and postpregnancy remission, unlike what was described in the study by Domingue et al., which reported a twofold greater remission rate in patients with microadenomas than in patients with macroadenomas (46% vs 26%) [26]. Contrary to what was previously described in the literature, our data showed a negative correlation between the number of pregnancies and the probability of disease remission; in fact, the women with the highest number of pregnancies had a lower healing rate. However, other studies have shown a similar percentage of patients in remission (18–33%) after a first, second or third pregnancy [26].

Finally, in women with prolactinoma, breastfeeding is generally considered safe, but few studies are available in the literature. In our study, no significant difference in remission rate was shown between women who breastfed and those who did not. Furthermore, breastfeeding duration was not related to remission status. These results, in agreement with the literature data, confirm that breastfeeding is not a risk factor for worsening of the disease [15]. Therefore, breastfeeding should not be discouraged in these patients.

The major limitation of our study is its retrospective nature. Moreover, the children were not prospectively followed up with formal developmental and language assessments, and their health data were exclusively reported by mothers during interviews. Therefore, it is not possible to entirely rule out the presence of recall bias. Furthermore, although the sample initially included a greater number of patients, the exclusion of some patients who were lost to follow-up or who refused to participate in the study decreased the sample size. To mitigate further reduction in cases number, statistical analyses were first conducted on the 58 pregnancies, each of which was treated as a distinct event. This approach neglected the potential familial predisposition to developmental disorders, potentially leading to an overestimation of the risk associated with the use of CAB. However, no substantial differences emerged analysing the first pregnancies only *(Table 3S).*

Finally, another limitation is the absence of a specific matched control group; therefore, the data concerning the occurrence of maternal-foetal and development outcomes were compared with those available for the general population.

## Conclusions

Foetal exposure to DA during the first weeks of embryogenesis is not associated with an increased risk of abortion, malformation or childhood disease. In our case series, a greater presence of mild and transient language disorders by maternal report was observed in the cabergoline group. However, it should be noted that this prevalence was comparable to that observed in the general population. Finally, our data did not currently allow us to consider pregnancy and breastfeeding as predictive factors for persistence or recovery from the disease.

### Supplementary Information

Below is the link to the electronic supplementary material.

Supplementary file1 (DOCX 24 KB)

## Data Availability

The data sets generated and/or analyzed during the current study are not publicly available but are avaiable from the corresponding author on reasonable request.
