# Supplementary material for: Prolactin-secreting tumors, dopamine agonists and pregnancy: a longitudinal experience of a tertiary neuroendocrine center
Source: Pituitary. 2024 Mar 18;27(3):269–76. doi: 10.1007/s11102-024-01384-1 (PMC11150308; doi:10.1007/s11102-024-01384-1)
Supplement: Supplementary file 1 — Supplementary file1 (DOCX 24 KB) [file 11102_2024_1384_MOESM1_ESM.docx]

**Table 1S**  Causes of caesarean section and complications during pregnancy or delivery

Abbreviation: ARDS = acute respiratory distress syndrome

|  | Cabergoline (n. 8) | Bromocriptine (n. 8) |
| --- | --- | --- |
| Caesarean section (n. 16)   - Breech presentation - Foetal macrosomia in gestational diabetes - Absence of valid contraction - Wrapping of umbilical cord - Mother’s advanced age - Foetal distress - Pre-eclampsia | 2  1  1  0  1  2  1 | 4  0  0  1  2  0  1 |
|  | Cabergoline (n. 6) | Bromocriptine (n. 7) |
| Complications during pregnancy or delivery (n. 13)   - Pre-eclampsia - Gestational diabetes - Previa placenta - Uterine fibroma - ARDS | 1  1  0  0  4 | 1  1  1  1  3 |

**Table 2S** Differences between clinical and biochemical data from patients with cabergoline- and bromocriptine-induced first pregnancy only.
Abbreviations: SD = standard deviation; IQR = interquartile range; DA = dopamine agonist; CAB = cabergoline; BRM = bromocriptine; ARDS = acute respiratory distress syndrome

| Variables | Pregnancies on Cabergoline  (n. 29) | Pregnancies on Bromocriptine  (n. 14) | P |
| --- | --- | --- | --- |
| Age at diagnosis (years); mean ± SD | 27.4 ± 6.7 | 25.9 ± 6.3 | 0.483 |
| Age at pregnancy (years); mean ± SD | 32.5 ± 5.7 | 31.1 ± 5.6 | 0.307 |
| Microadenoma n; (%) | 24 (82.7%) | 9 (64.3%) | 0.337 |
| Adenoma maximum diameter at diagnosis (mm); median [IQR] | 6 [5.8; 8] | 6.5 [5; 12] | 0.834 |
| Prolactin levels at diagnosis (ng/ml); median [IQR] | 107.9 [64.2; 139] | 112.3 [37; 288] | 0.593 |
| Adenoma diameter nadir before pregnancy (mm); mean ± SD | 5.4 ± 3 | 7.4 ± 3.4 | 0.098 |
| Prolactin levels nadir before pregnancy (ng/ml); median [IQR] | 11.4 [4.5; 24.4] | 4.9 [3.5; 12.5] | 0.365 |
| Breastfeeding yes; n (%) | 16 (55%) | 7 (50%) | 0.798 |
| Breastfeeding weeks; median [IQR] | 16 [1.5; 37] | 4 [0;16] | 0.072 |
| DA foetal exposure (days); median [IQR] | 28 [28;35] | 31.5 [28;35] | 0.742 |
| Preterm deliveries (n) | 2 | 6 | 0.812 |
| Child weight at birth (gr); median [IQR] | 3450 [3165; 3715] | 3350 [3040; 3587] | 0.623 |
| Child born underweight; n (%) | 3 (10.3%) | 0 (0%) | 0.560 |
| Child born with macrosomia; n (%) | 1 (3%) | 1 (7%) | 0.834 |
| APGAR Score; median [IQR] | 9 [9; 9] | 9 [9; 9] | 0.698 |
| Child gender male; n (%) | 18 (62%) | 7 (50%) | 0.767 |
| Persistence of disease; n (%) | 17 (58.6%) | 7 (50%) | 0.740 |
| Adenoma diameter at last follow up (mm); median [IQR] | 2 [0;5.0] | 3 [1.9;5.4] | 0.258 |
| Prolactin levels at last follow up (ng/ml); median [IQR] | 14.6 [8.3; 39.5] | 21.5 [5.4; 48.1] | 0.566 |
| Delivery/pregnancy complications (yes); n (%) | 10 (34.5%) | 1 (7.1%) | 0.148 |
| Child complications (yes); n (%) | 7 (24%) | 0 (0%) | 0.084 |
| Child speech disorders (yes); n (%) | 6 (20.7%) | 0 (0%) | 0.222 |

**Table 3S** Variables associated with developmental disorders considering first pregnancies only

Abbreviations: IQR = interquartile range; SD = standard deviation; DA = dopamine agonist, CAB = cabergoline; BRM = bromocriptine

| Variables | No developmental disorders  (n. 35) | Developmental disorders  (n. 6) | P value |
| --- | --- | --- | --- |
| Age at diagnosis; mean ± SD | 26.6 ± 6.0 | 29.0 ± 7.1 | 0.351 |
| Age at pregnancy; mean ± SD | 31.8 ± 5.7 | 33.0 ± 7.0 | 0.645 |
| Microadenoma; n (%) | 27 (77%) | 5 (83%) | 0.845 |
| Adenoma maximum diameter at diagnosis (mm); median [IQR] | 7 [5.0 - 8.7] | 6 [6.0-6.0] | 0.682 |
| Prolactin levels at diagnosis (ng/ml); median [IQR] | 121 [69.4-187.9] | 65 [48.6-87.5] | 0.051 |
| Adenoma diameter nadir before pregnancy (mm); median [IQR] | 6.0 [5.0-8.0] | 5.0 [0.0-6.0] | 0.111 |
| Prolactin levels nadir before pregnancy (ng/ml); median [IQR] | 9.0 [4.5-21.4] | 4.5 [0.9-19.4] | 0.280 |
| Breastfeeding (yes); n (%) | 19 (54%) | 4 (67%) | 0.904 |
| Breastfeeding duration (weeks); median [IQR] | 8 [0-25] | 11 [0-36] | 0.708 |
| Foetal Exposure to DA (days); median [IQR] | 28 [28-35] | 31 [28-35] | 0.953 |
| CAB; n (%) BRM; n (%) | 23 (65.7%)  12 (34.3%) | 6 (100%)  0 (0%) | 0.222 |
| Child weight at birth (gr); median [IQR] | 3350 [3010-3700] | 3550 [3370- 3680] | 0.288 |
| Delivery/pregnancy complications (yes); n (%) | 9 (26%) | 2 (33%) | 0.937 |
| APGAR SCORE; median [IQR] | 9 [9-9] | 9 [9-9] | 0.246 |
| APGAR SCORE  ≤ 6  7  8  9  10 | 0  1  1  19  5 | 0  0  1  5  0 | 0.381 |

**Mother interview script:**

1. Did any issues arise during routine gynecological/obstetric check-ups throughout the pregnancy? If so, what were they?

2. Did you have a full-term delivery? If not, for what reason and at what gestational week did you deliver?

3. Was the delivery spontaneous or by cesarean section? Were there any complications during delivery? If so, what were they?

4. Were there any issues noted during the immediate postpartum period at the neonatal examination?

5. Could you provide the birth weight and APGAR score?

6. Did you breastfeed? If so, for how long?

7. During these years, has your child undergone routine pediatric evaluations during the neonatal period and subsequently during development? Were any issues identified during these medical assessments? If so, what were they?
